# NT-pro BNP and plasma-soluble ST2 as promising biomarkers for hypertension, hypertensive heart disease and heart failure in sub-Saharan Africa

**Published:** 2017

**Authors:** Dzudie Anastase, Dzekem Suiru, Pascal Kengne Andre

**Affiliations:** Department of Medicine, University of Cape Town, Cape Town, South Africa; Douala General Hospital and Clinical Research Education Networking and Consultancy, Douala, Cameroon; Faculty of Health Sciences, University of Buea, Buea, Cameroon; Douala General Hospital, and Clinical Research Education Networking and Consultancy, Douala, Cameroon; Faculty of Health Sciences, University of Buea, Buea, Cameroon; Department of Medicine, University of Cape Town, and Non-Communicable Diseases Research Unit, South African Medical Research Council, Cape Town, South Africa

Dear Sir

In three recent publications,^1^-^3^ Ojji and colleagues reported on the role of two novel biomarkers, NT-pro-BNP and plasma soluble ST2, for differentiating sub-Saharan African people with hypertension (HT) without left ventricular hypertrophy (LVH) and without heart failure (HF) from hypertensive people with LVH (HTLVH) and those with HF (HTHF). The authors clinically and echocardiographically evaluated a group of 210 patients with hypertension residing in Abuja, the capital city of Nigeria. All these patients had measurements done for the cardiac neurohormone NT-pro-BNP and for soluble ST2, which is a novel cardiac biomarker of mechanical strain.

In the first publication,^1^ in which they investigated the effect of LV remodelling on the concentration of soluble ST2, the authors found that subjects with HTHF had higher plasma ST2 concentrations compared to those with HTLVH and those with HT (134.7 ± 57.3 vs 23.0 ± 8.3 vs 14.5 ± 4.9 ng/ml, all p < 0.0001). Soluble ST2 also had a strong correlation with clinical and echocardiographic parameters. The authors concluded that ‘Plasma ST2 is a useful biomarker in not only differentiating HTHF from HT with or without LVH, but also distinguishes hypertensive LVH from HT without LVH’.

In the second publication,^2^ the authors investigated the relationship between soluble ST2 levels and LV geometric patterns in the same cohort of patients with HT and found that patients with concentric LVH had higher soluble ST2 levels compared with patients with normal LV geometry (20.4 ± 8.4 vs 14.3 ± 5.4 ng/ml, p < 0.002). This also led to the conclusion that ‘soluble ST2 level is not only affected by hypertensive LVH, but may be a future biomarker in differentiating concentric hypertrophy from normal geometry in hypertension’.

In the third study,3 the authors examined the effect of NT-pro- BNP on LV and RV remodelling in this same hypertensive African cohort. Participants with HTHF had significantly higher NT-pro-BNP levels compared to those with HTLVH. Based on these results, the authors proposed that NT-pro-BNP could be a useful biomarker for differentiating HT with or without LVH from HTHF in black hypertensive subjects.

The conclusions drawn by these authors are valid exclusively in the study context for a number of reasons. First, it is important to remember that hypertension and hypertensive heart disease, which are potentially preventable diseases, are the main contributors of the growing burden of heart failure in SSA.^4^ Second, echocardiography is globally the cornerstone of the routine assessment of various types of hypertensive heart disease as it allows for the detection of normal left ventricular concentric remodelling, concentric versus eccentric LVH, and HF with the possible differentiation between HF with reduced ejection fraction and HF with preserved ejection fraction using tissue Doppler imaging, as well as the measurement of pulmonary artery pressure, another prognostic marker in this population.^5^ However, echocardiography remains expensive, less available in most SSA settings, and requires experts both for its performance and interpretation. For these reasons, requesting cardiac echocardiography in most settings in SSA is like searching for the goose that lays the golden egg.

There is value therefore in using circulating biomarkers, which could be useful as surrogate markers of the heart disease process in resource-poor settings. Natriuretic peptide (BNP and NT-pro- BNP) levels have been shown to accurately reflect left ventricular pressure, and studies have found that peptide levels are sensitive and specific for diagnosing heart failure and also relevant for risk stratification.^6^,^7^ There is no doubt that the third study by Ojji et al.^3^ is a confirmation of the usefulness of the diagnostic role of NT-pro BNP in the SSA setting and it has immediate relevance for clinicians.

Contrary to NT-pro BNP measurement, which is already an established gold standard for HF, soluble ST2 as a biomarker has been less investigated. Just as with other novel biomarkers, such as mid-regional pro-atrial natriuretic peptide (MR-proANP) and galectin-3, which are promising diagnostic and prognostic biomarkers beyond established natriuretic peptides, the role of soluble ST2 in the clinical care of patients is yet to be established beyond any doubt. It is very encouraging to note that, as suggested by Ojji and co-workers, soluble ST2 can help differentiate HT, HTLVH and HTHF, as well as concentric LVH from normal LV geometry.

The relevance of these three studies by Ojji and co-workers in this particular context should however not completely overshadow the shortcomings. First, this was a single-centre study with a limited number of participants. Second, heart disease and especially heart failure in this population may have included cases of different severity and chronicity, especially ischaemic causes, which could limit the generalisability of their findings to all hypertensive patients. Finally, further research is required to determine the optimal cut-off points for diagnosis, the relevance of serial measurements, changes following treatment, and the prognostic role in Africans, before they can be widely recommended for clinical decision making.
